# The size of retrieved lymph nodes correlates with the number of retrieved lymph nodes and is an independent prognostic factor in patients with stage II colon cancer

**DOI:** 10.1007/s00384-015-2357-9

**Published:** 2015-08-12

**Authors:** Kazutake Okada, Sotaro Sadahiro, Toshiyuki Suzuki, Akira Tanaka, Gota Saito, Shinobu Masuda, Yasuo Haruki

**Affiliations:** Department of Surgery, Tokai University, 143 Shimokasuya Isehara, Kanagawa, 259-1193 Japan; Department of Pathology, Nihon University, Tokyo, Japan; Department of Basic Medical Science, Tokai University, Isehara, Japan

**Keywords:** Colonic neoplasm, Adenocarcinoma, Lymph nodes, Survival analysis, Retrospective study

## Abstract

**Purpose:**

In stage II colon cancer, patients with many retrieved lymph nodes (LNs) have been reported to have better oncological outcomes. We tested the hypothesis that the greater number of retrieved LNs is related to a larger LN size.

**Methods:**

The subjects comprised 320 patients with stage II colon cancer who underwent curative resection. All operations were elective and were performed by the same surgeons. The maximum long axis and short axis diameters of LNs were measured on hematoxylin-eosin-stained specimens.

**Results:**

A total of 4,744 LNs were evaluated. The number of retrieved LNs was 14.8 ± 10.1 (mean ± SD). The long axis diameter was 4.8 ± 2.6 mm, with a median value of 4.3 mm, a maximum value of 20.4 mm, and a minimum value of 0.6 mm. The corresponding short axis diameters were 3.4 ± 1.7, 3.0, 15.1, and 0.5 mm, respectively. The highest correlation coefficient for the association with the number of LNs was obtained for the maximum value of the long axis diameter (0.59). Multivariate analysis revealed that age, tumor location, pathological T stage, and the maximum long axis diameter were independent prognostic factors. The number of LNs was not a significant factor. Patients with less than 12 LNs and a maximum long axis diameter of less than 10 mm had significantly poorer outcomes (*p* < 0.001).

**Conclusion:**

In patients with stage II colon cancer, the maximum long axis diameter of LNs correlated with the number of LNs and was an independent prognostic factor.

## Introduction

Low numbers of retrieved lymph nodes (LNs) have been linked to poor outcomes in patients with stage II or III colon cancer [1–7]. The Working Party Report to the World Congress of Gastroenterology in Sydney in 1990 recommended that at least 12 LNs are examined to ensure accurate staging of disease [8]. Inadequately sampled LNs are a high risk factor in stage II colon cancer, and several guidelines have recommended that postoperative adjuvant chemotherapy should be considered in such patients [9, 10].

In Japan, LNs are collected by surgeons immediately after surgery. LNs are macroscopically examined and removed from the resected mesentery, fixed in formalin, and submitted for histopathological examination. Even if the extent of LN dissection is similar, the number of retrieved LNs differs among patients. We frequently encounter patients with large LNs that can be easily identified and recovered, as well as those with small LNs that are difficult to find even on careful examination. The present study was performed to test the hypothesis that a greater number of retrieved LNs are related to a larger LN size. We also examined factors influencing the number of retrieved LNs and outcomes in patients with stage II colon cancer.

## Patients and methods

### Patients

Among patients who underwent elective radical surgery in Tokai University Hospital from January 1991 through December 2003, we studied 320 patients with pathological stage II colon cancer. Patients who underwent emergency surgery were excluded from the present study. The median follow-up time for living patients was 9.8 years (range, 8.3 to 11.6). This is a retrospective chart review of a prospectively maintained database.

### Surgery

All operations were performed by four or five staff members consisting of two or three colorectal staff surgeons (SS, TS, AT, KO, or GS) and one or two members of the surgical team. All procedures were open surgery, and no patient underwent laparoscopic surgery. All pericolic nodes, intermediate nodes, and main nodes were dissected [11]. LN dissection was distally extended to the bifurcation of the ileocolic artery or right colic artery (or both) from the superior mesenteric artery in patients with right-sided colon cancer, the bifurcation of the middle colic artery from the superior mesenteric artery in patients with transverse colon cancer, and the bifurcation of the inferior mesenteric artery from the aorta in patients with left-sided colon cancer. Both the distal and proximal resection margins were at least 5 cm from the tumor margin.

### Pathological procedures

One member of the surgical team pinned the resected specimen to a corkboard and identified the blood vessels. The mesentery was classified into three regions: the pericolic lymph node region, the intermediate lymph node region, and the main lymph node region. The lymph nodes were retrieved from each region and were placed in separate containers and submitted to the pathological department. LNs were identified by direct inspection and manual palpation after closely slicing the mesocolon. Fat clearance methods were not used in any patient. Pathologists examined all specimens considered candidate LNs. LNs fixed in formalin were sliced to obtain the maximal cut surface and were stained with hematoxylin and eosin.

### Evaluation of the numbers and sizes of retrieved LNs

The numbers of retrieved LNs were obtained by reviewing the patients’ pathological charts. Pathological slides were prepared with the use of a digital camera, and LN size (longest axis, shortest axis) was measured using a computer digitizer (Adobe Photoshop CS5®, Adobe Systems, San Jose, CA, USA, ImageJ 1.47, National Institutes of Health, Bethesda, MD, USA). The mean, median, maximum, and minimum values of the longest and shortest axis diameters of LNs were calculated for each patient.

### Clinicopathological variables examined

In addition to LN size, we examined the relations of the following clinicopathological factors to the number of retrieved LNs and outcomes: sex, age, tumor location, pathological T stage, histological type, lymphatic invasion, and venous invasion.

### Statistical analyses

Correlations of the number of retrieved LNs with LN size were evaluated with the use of Pearson correlation coefficients. When examining the relations between the number of retrieved LNs and clinicopathological variables, the number of retrieved LNs was separately analyzed as a continuous variable as well as a categorical variable (<12 vs. ≥12). Groups were compared with the use of the Fisher’s exact test or the chi-square test for categorical variables and the Mann-Whitney *U* test or the Kruskal-Wallis test for continuous variables.

Cancer outcomes evaluated included overall survival (OS) at the time of the patient’s last follow-up. Cumulative survival rates were calculated by the Kaplan-Meier method, and differences between groups were tested with the use of the log-rank test. To determine the optimal cutoff value of LN size for survival analysis, cutoff values were tentatively set at 2-mm intervals. Patients were then divided into two groups according to the tentative cutoff values: those with LNs smaller than the cutoff value and those with LNs equal to or greater than the cutoff value. Overall survival was then compared between each of the groups. The cutoff value associated with the smallest *p* value was defined as the optimal cutoff value.

Cox proportional hazards modeling was used to adjust comparisons for the clinicopathological variables described above. The numbers of retrieved LNs and LN size were considered continuous variables.

In all statistical analyses, a two-sided value of *p* < 0.05 was considered to indicate statistical significance. Statistical calculations were performed using JMP ver. 11 software (SAS Institute Inc., Cary, NC, USA).

This study was approved by the institutional review board of our university (08R-032).

## Results

### Patients’ characteristics

The patients’ characteristics are summarized in Table 1. A total of 320 patients (123 women) were studied. The mean age at the time of surgery was 64.8 ± 12.2 years (mean ± standard deviation). The most common tumor location was the sigmoid colon/rectosigmoid colon (54 % of patients). Pathological T stage was classified as T3 in 82 % of the patients and T4 in 18 %.

**Table 1:** Patient’s characteristics (*n* = 320)

Variable	*n* (%)
Sex	
Male	197 (62)
Female	123 (38)
Age	
Mean ± SD	64.8 ± 12.2
Quartiles	57, 66, 74
Location of the tumor	
Cecum	32 (10)
Ascending colon	54 (17)
Transverse colon	42 (13)
Descending colon	19 (6)
Sigmoid colon/rectosigmoid colon	173 (54)
Pathological T stage	
T3	263 (82)
T4	57 (18)
Histological type	
Well	185 (58)
Moderate	116 (36)
Poor	19 (6)
Lymphatic invasion	
Positive	261 (82)
Negative	59 (18)
Venous invasion	
Positive	226 (71)
Negative	94 (29)

*Well* well-differentiated adenocarcinoma, *Moderate* moderately differentiated adenocarcinoma, *Poor* poorly differentiated adenocarcinoma

### Numbers and sizes of retrieved LNs

A total of 4,744 LNs were evaluated. Table 2 shows the numbers and sizes of retrieved LNs. The mean number of retrieved LNs was 14.8 ± 10.1, with a median value of 12.0. The number of retrieved LNs was less than 12 in 149 patients (47 %) and 12 or more in 171 (53 %). The mean long axis diameter of LNs was 4.80 ± 2.59 mm, with a median value of 4.3 mm, a maximum value of 20.4 mm, and a minimum value of 0.6 mm. The mean short axis diameter of LNs was 3.36 ± 1.71 mm, with a median value of 3.0 mm, a maximum value of 15.1 mm, and a minimum value of 0.5 mm.

**Table 2:** Numbers and sizes of retrieved lymph nodes (320 patients)

Number of retrieved LNs	
Mean ± SD	14.8 ± 10.1
Quartiles	8.0, 12.0, 20.0
<12 nodes	149 (47 %)
≥12 modes	171 (53 %)
Long axis (mm) (*n* = 4,744)	
Mean ± SD	4.80 ± 2.59
Quartiles	3.0, 4.3, 6.0
Maximum	20.4
Minimum	0.6
Short axis (mm) (*n* = 4,744)	
Mean ± SD	3.36 ± 1.71
Quartiles	2.1, 3.0, 4.3
Maximum	15.1
Minimum	0.5

### Relation between the number of retrieved LNs and the size of the LNs

Table 3 shows the relation between the number of retrieved LNs and the size of LNs. The correlation coefficient for the association between the number of retrieved LNs and the long axis diameter of the nodes was 0.23 for the mean long axis diameter, 0.16 for the median value, 0.59 for the maximum value, and −0.29 for the minimum value. The correlation coefficient for the association between the number of retrieved LNs and the short axis diameter of the nodes was 0.18 for the mean value, 0.13 for the median value, 0.54 for the maximum value, and −0.33 for the minimum value. For both the long axis and short axis diameters, the highest absolute correlation coefficients for the association with the number of retrieved nodes were obtained for the maximum values, indicating a moderately positive correlation.

**Table 3:** Relation between the number of retrieved lymph nodes and the size of the nodes

		Long axis				Short axis			
		Mean	Median	Maximum	Minimum	Mean	Median	Maximum	Minimum
Number of retrieved LNs	*r*	0.23	0.16	0.59	−0.29	0.18	0.13	0.54	−0.33
	*p* value	<0.01	<0.01	<0.01	<0.01	<0.01	0.02	<0.01	<0.01

*r* Pearson’s correlation coefficient

**Fig. 1:**
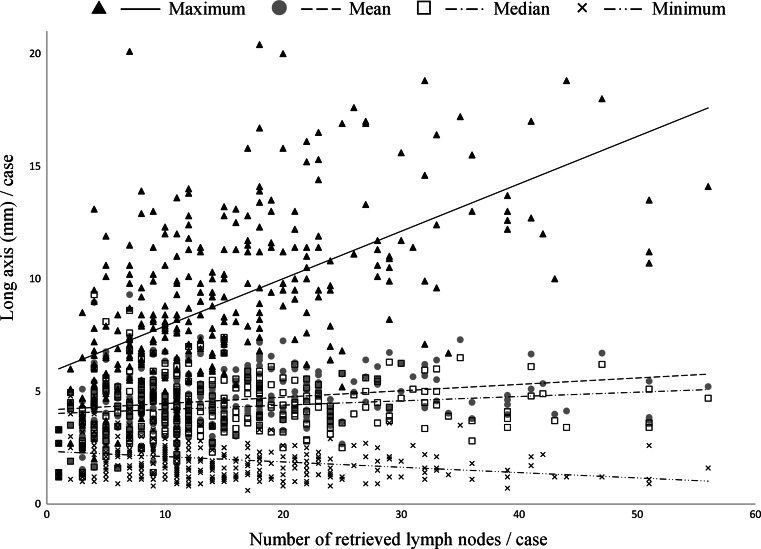
Relation between the number of retrieved lymph nodes and the long axis. The numbers of retrieved lymph nodes are plotted against the mean, median, maximum, and minimum long axis diameters of the lymph nodes (*scatter plot*). The *straight lines* are linear regression lines showing the relations between each variable and the number of retrieved lymph nodes

As the number of retrieved LNs increased, the maximum long axis diameter became greater, the minimum long axis diameter became smaller, and the dispersion of the values increased. The mean and median diameters of LNs showed virtually no correlation with the number of retrieved LNs (Fig. 1). On the basis of these results, the maximum long axis diameter was used as a representative value of LN size.

### Number of retrieved LNs according to selected variables

Table 4 shows the relations between clinicopathological factors and the number of retrieved LNs. An age of younger than 65 years and tumors located in the cecum or ascending colon were significantly associated with a greater number of retrieved LNs and a higher proportion of patients with 12 or more retrieved LNs. When the maximum long axis diameter of LNs was classified at 5-mm intervals, an incremental increase in the maximum long axis diameter was accompanied by a stepwise increase in the number of LNs and the proportion of patients with 12 or more retrieved LNs.

**Table 4:** Number of retrieved lymph nodes by selected variables (320 patients)

Variable	Number of retrieved LNs Mean ± SD	*p* value	Patients with ≥12 LNs total 171 (53 %)	*p* value
Sex				
Men	14.5 ± 10.1	0.500^a^	100/197 (51)	0.250^c^
Women	15.3 ± 10.3		71/123 (58)	
Age (year)				
<65	16.8 ± 10.5	<0.001^a^	86/138 (62)	0.007^c^
≥65	13.4 ± 9.6		85/182 (47)	
Location of the tumor				
Cecum	17.8 ± 11.2	<0.001^b^	23/32 (72)	<0.001^d^
Ascending colon	20.4 ± 11.1		43/54 (80)	
Transverse colon	15.9 ± 11.6		24/42 (57)	
Descending colon	10.5 ± 8.9		5/19 (26)	
Sigmoid colon/rectosigmoid colon	12.7 ± 8.5		76/173 (44)	
Pathological T stage				
T3	14.8 ± 10.0	0.855^a^	140/263 (53)	0.885^c^
T4	14.9 ± 11.0		31/57 (54)	
Histologic type				
Well	14.5 ± 10.2	0.542^b^	96/185 (52)	0.627^d^
Moderate	15.4 ± 10.5		63/116 (54)	
Poor	15.1 ± 7.6		12/19 (63)	
Lymphatic invasion				
Positive	14.5 ± 10.0	0.228^a^	136/261 (52)	0.386^c^
Negative	16.2 ± 10.9		35/59 (59)	
Venous invasion				
Positive	14.3 ± 9.4	0.322^a^	119/226 (53)	0.713^c^
Negative	16.2 ± 11.6		52/94 (55)	
Maximum long axis diameter of LNs (mm)				
<5.0	5.6 ± 4.1	<0.001^b^	3/37 (8)	<0.001^d^
5.0–9.9	12.2 ± 6.7		76/172 (44)	
10.0–14.9	20.8 ± 11.7		71/89 (80)	
≥15	27.0 ± 9.6		21/22 (95)	

^a^Mann-Whitney *U* test

^b^Kruskal-Wallis test

^c^Fisher’s exact test

^d^Chi-square test

### Overall survival according to the number of LNs and the maximum long axis diameter of LNs

**Fig. 2:**
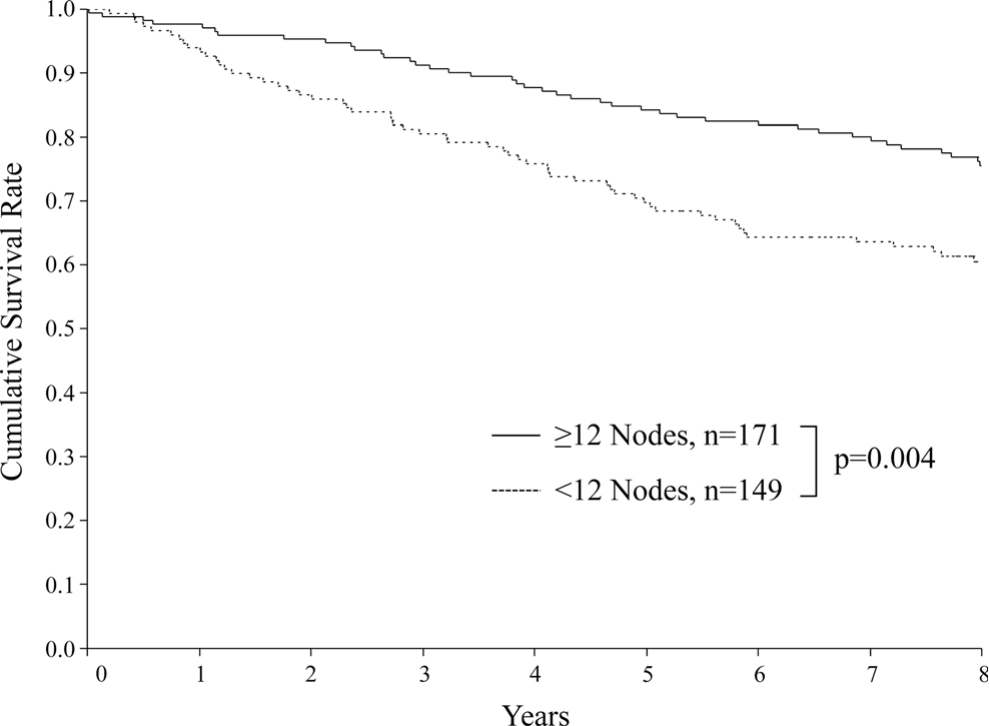
Overall survival according to number of retrieved lymph nodes

In patients with 12 or more retrieved LNs, the overall survival rate was 84 % at 5 years and 76 % at 8 years, which was significantly better than the corresponding rates in patients with less than 12 retrieved LNs (*p* = 0.004) (Fig. 2).

The optimal cutoff value for the maximum long axis diameter of LNs was set at 10 mm because the *p* value was smallest (Table 5). This value was used for analysis. Patients in whom the maximum long axis diameter of LNs was more than 10 mm had a 5-year survival rate of 84 % and showed a trend toward better outcomes than those in whom the maximum long axis diameter of LNs was less than 10 mm (74 %; *p* = 0.055) (Table 5).

**Table 5:** Kaplan-Meier 5-year overall survival according to maximum long axis diameter of lymph nodes

Cutoff value (mm)	Maximum long axis diameter of LNs (mm)	*n*	5-year OS %	*p* ^a^
6	<6	65	74	0.607
	≥6	255	78	
8	<8	148	74	0.132
	≥8	172	81	
10	<10	209	74	0.055
	≥10	111	84	
12	<12	260	76	0.488
	≥12	60	83	
14	<14	293	77	0.367
	≥14	27	85	

^a^Log-rank test

**Fig. 3:**
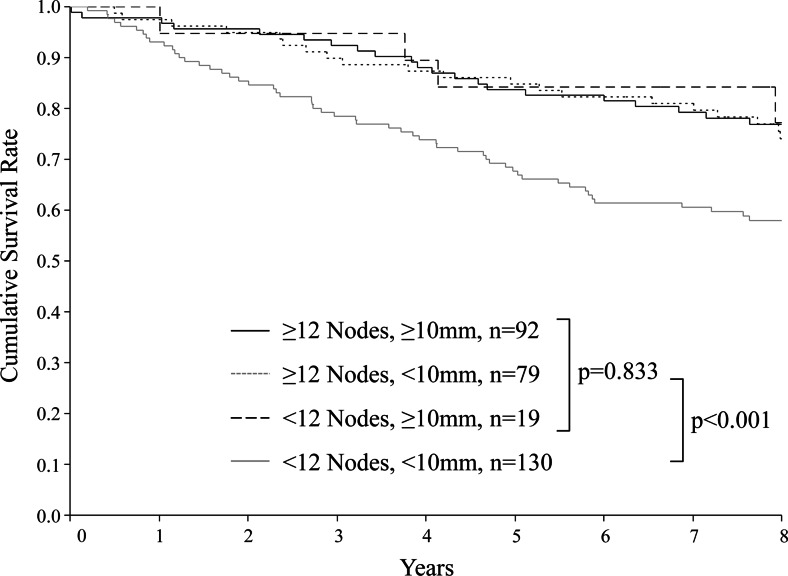
Overall survival according to number of lymph nodes and maximum long axis diameter of lymph nodes

The patients were divided into four groups according to two factors: whether the number of retrieved LNs was <12 or ≥12 and whether the maximum long axis diameter was <10 or ≥10 mm. Overall survival was compared among the four groups (Fig. 3). Patients with <12 retrieved LNs and a maximum long axis diameter of <10 mm had significantly poorer outcomes, with an overall survival rate of 68 % at 5 years and 58 % at 8 years (*p* < 0.001). Although the number of patients was small (*n* = 19), patients who had <12 retrieved LNs and a maximum long axis diameter of ≥10 mm had an overall survival rate of 84 % at 5 years and 77 % at 8 years, which did not differ from the survival rates of patients with ≥12 retrieved LNs.

### Multivariate Cox model of overall survival (Table 6)

**Table 6:** Multivariable Cox model for overall survival

Characteristic	HR	95 % CI	*p* value	HR	95 % CI	*p* value
Sex						
Men	1.0	Reference		1.0	Reference	
Women	0.81	0.54–1.22	0.315	0.86	0.57–1.29	0.473
Age (year)	1.04^a^	1.02–1.06	<0.001	1.04^a^	1.02–1.06	<0.001
Location of the tumor						
Cecum	2.05	1.07–3.69	0.030	2.42	1.24–4.48	0.011
Ascending colon	1.46	0.82–2.53	0.199	1.41	0.81–2.37	0.218
Transverse colon	1.39	0.73–2.47	0.296	1.36	0.72–2.42	0.323
Descending colon	0.90	0.34–2.00	0.812	0.86	0.32–1.91	0.729
Sigmoid colon/rectosigmoid colon	1.0	Reference		1.0	Reference	
Pathological T stage						
T3	1.0	Reference		1.0	Reference	
T4	3.30	2.12–5.04	<0.001	3.59	2.29–5.54	<0.001
Histological type						
Well	1.0	Reference		1.0	Reference	
Moderate	1.00	0.66–1.49	0.986	0.97	0.65–1.46	0.894
Poor	0.55	0.16–1.36	0.214	0.58	0.17–1.43	0.259
Lymphatic invasion						
Positive	1.0	Reference		1.0	Reference	
Negative	0.93	0.51–1.60	0.792	0.95	0.52–1.64	0.856
Venous invasion						
Positive	1.0	Reference		1.0	Reference	
Negative	0.76	0.47–1.19	0.237	0.73	0.45–1.14	0.169
Number of retrieved LNs	0.98^b^	0.96–1.00	0.118			
Maximum long axis diameter of LNs (mm)				0.93^c^	0.88–0.99	0.021

^a^Estimates the ratio of a 1-year incremental increase in age

^b^Estimates the ratio of a one-node incremental increase in the number of LNs

^c^Estimates the ratio of a 1-mm incremental increase in lymph node size

The results obtained using a multivariate Cox model of overall survival are shown in Table 6. The number of retrieved LNs was related to the maximum long axis diameter of LNs. Therefore, these variables were separately evaluated to avoid multicollinearity. Age at the time of surgery, tumor location, pathological T stage, and the maximum long axis diameter of LNs were independent prognostic factors. The number of retrieved LNs was not a significant factor. Higher age, cecal tumors, and T4 tumors were associated with poorer overall survival. A greater maximum long axis diameter of LNs was associated with better overall survival. When the number of retrieved LNs was analyzed as a categorical variable, it became an independent predictive factor (data not shown).

## Discussion

Previous studies have reported that the number of retrieved LNs is related to oncological outcomes in patients with colorectal cancer without distant metastasis [1–7]. The tumor-node-metastasis classification of the Union for International Cancer Control (UICC) and the American Joint Committee on Cancer (AJCC) recommends that at least 12 LNs are examined to accurately evaluate N stage [12, 13].

In our study, the overall survival rate was lower in patients with <12 retrieved LNs than in those with ≥12 retrieved LNs. However, various cutoff values have been recommended for the number of retrieved LNs required to accurately evaluate N stage, including 7 or more [14], 8 or more [15], 10 or more [16], 13 or more [4, 17], 14 or more [18], 15 or more [6], 17 or more [1], 18 or more [19, 20], and 20 or more [3]. The optimal cutoff value for the number of retrieved LNs thus remains controversial.

In colon cancer, the number of retrieved LNs is influenced by patients’ factors, surgical factors, and pathological factors. In previous studies, such factors included the patient’s age, year of diagnosis, anatomic site, specimen length, tumor size, pathological T stage, tumor grade of differentiation, LN status, and surgeon [21–23]. In our study, the number of retrieved LNs was influenced by age, tumor location, and the maximum long axis diameter of the retrieved nodes.

The lower number of LNs in elderly patients has been attributed to the smaller range of LN dissection in elderly patients than in younger adults and the age-related regression of LNs [24–26]. The higher number of LNs retrieved in right-sided colon cancer than in left-sided colon cancer has been ascribed to the proliferation of lymphatic tissue around the ileocecal region and the longer resected length of the right side of the colon than the left side [27]. The length of the resected bowel might affect the number of retrieved LNs. West et al. reported that the lengths of the resected colon in Germany were significantly longer than those in Japan, even if the tumor location was right-sided, transverse, or left-sided. In addition, the number of retrieved LNs was significantly greater in Germany than in Japan [28]. Therefore, in the future, the appropriate number of retrieved lymph nodes might be determined on the basis of patients’ age, sex, resected site, and length of the resected bowel.

Few studies have evaluated the relation between the number of retrieved LNs and LN size. Sloothaan et al. reported recently that the median value of LN size is associated with the number of retrieved LNs in patients without LN metastasis [29]. Markl et al. reported that the total long axis diameter of retrieved LNs correlates with the number of retrieved LNs [30]. In general, however, the sum of the long axis diameter increases in parallel to the number of retrieved nodes. In the present study, the number of retrieved LNs positively correlated with the maximum long axis diameter of LNs. The reason why the mean and median long axis diameters of LNs did not correlate with the number of retrieved LNs may be that the numbers of small as well as large LNs increased with a greater number of retrieved LNs, leading to a greater dispersion of LN size (Fig. 1).

The association between few retrieved LNs and poor outcomes has been suggested to be related to understaging and tumor immunity [5, 14]. LN metastases have been detected even in small LNs less than 5 mm in diameter [30–35]. Because outcomes are unaffected by the size of metastatic LNs [36], it is important to examine even small LNs 1 to 2 mm in diameter [1]. However, the risk of tumor understaging caused by overlooking small LNs less than 3 mm in diameter and less than 5 mm in diameter has been estimated to be 1.3 % [29] and 2 to 5 % [30], respectively. This value is considered too small to account for the fact that a low number of retrieved LNs (i.e., understaging) is associated with poor outcomes. Factors other than staging accuracy may account for the improvement in survival associated with increased numbers of LNs evaluated in patients with colon cancer [7].

The relation between tumor immunity and the number of retrieved LNs remains unclear. In colorectal cancer, high numbers of tumor-infiltrating lymphocytes are associated with increased numbers of retrieved LNs [37], as well as with better outcomes [38–42]. However, few studies have evaluated the relation between the size of retrieved LNs and outcomes.

The shrinkage rate of lymph nodes after fixation in formalin and staining with hematoxylin-eosin has been reported to be 10 to 16 % regardless of metastases [43, 44]. Therefore, 10 mm on hematoxylin-eosin-stained pathological slides corresponds to 11 to 12 mm in vivo.

Murphy et al. reported that patients in whom the mean long axis diameter of retrieved LNs was <4 mm had poorer outcomes than those in whom the mean long axis diameter was ≥4 mm [26]. Märkl et al. reported that the retrieval of seven or more LNs with a long axis diameter of ≥5 mm was associated with better outcomes than the retrieval of less than seven LNs of the same size in patients with stage I or II colon cancer [30]. In our study, when the number of retrieved LNs was less than 12, a maximum long axis diameter of <10 mm for the retrieved nodes was associated with poor outcomes. In patients with a maximum long axis diameter of ≥10 mm, outcomes were better, regardless of the number of retrieved LNs.

Apart from tumor metastasis, an increase in LN size can be caused by hyperplasia of cellular components in LNs [45]. In colon cancer, follicular hyperplasia can occur in enlarged regional LNs without metastasis [30]. Therefore, in stage II colon cancer without LN metastasis, the size of regional LNs may reflect the immune status of patients and cancer-specific immune responses [5, 21, 26, 30, 46, 47].

Our results showed that the maximum long axis diameter of LNs was an independent prognostic factor in patients with stage II colon cancer unaccompanied by LN metastasis. A larger maximum long axis diameter of LNs was associated with better outcomes, regardless of the number of retrieved LNs. Although the number of retrieved LNs is influenced by the length of the resected intestine and the extent of dissection, the maximum long axis diameter of LNs is unlikely to be affected by these factors.

In conclusion, the maximum long axis diameter of retrieved LNs correlated with the number of retrieved LNs and was an independent prognostic factor in patients with stage II colon cancer. A prospective multicenter trial is needed to confirm the clinical significance of these variables as predictors of oncological outcomes.
